# Estrogen receptor-mediated miR-486-5p regulation of OLFM4 expression in ovarian cancer

**DOI:** 10.18632/oncotarget.7236

**Published:** 2016-02-07

**Authors:** Hanyu Ma, Tian Tian, Shuang Liang, Xubin Liu, Hongwei Shen, Meng Xia, Xingyang Liu, Wenhui Zhang, Liantang Wang, Shangwu Chen, Li Yu

**Affiliations:** ^1^ Department of Pathology, The First Affiliated Hospital, Sun Yat-sen (Zhongshan) University, Guangzhou, China; ^2^ Department of Gynecology and Obstetrics, The First Affiliated Hospital, Sun Yat-sen (Zhongshan) University, Guangzhou, China; ^3^ State Key Laboratory for Biocontrol, Guangdong Key Laboratory of Pharmaceutical Functional Genes, Department of Biochemistry, School of Life Sciences, Sun Yat-sen (Zhongshan) University, Guangzhou, China

**Keywords:** OLFM4, ovarian serous adenocarcinoma, estrogen, miR-486-5p

## Abstract

Estrogen signaling influences the development and progression of ovarian tumors, but the underlying mechanisms are not well understood. In a previous study we demonstrated that impairment of estrogen receptor alpha (ERα)-mediated olfactomedin 4 (OLFM4) expression promotes the malignant progression of endometrioid adenocarcinoma, and we identified OLFM4 as a potential target of miR-486-5p. In this study we investigated the role of OLFM4 in ovarian serous adenocarcinoma. Ovarian serous adenocarcinoma tissues had reduced OLFM4 expression. Expression of OLFM4 was positively correlated with ERα expression, and estrogen (E2) treatment in ovarian cancer cells induced OLFM4 expression in an ERα-dependent manner. In contrast to ERα, miR-486-5p levels were inversely correlated with OLFM4 expression in ovarian serous adenocarcinoma. Ovarian cancer cells transfected with miR-486-5p mimics showed decreased *OLFM4* mRNA expression, and ovarian cancer cells treated with E2 showed reduced cellular miR-486-5p levels. *OLFM4* knockdown enhanced proliferation, migration, and invasion by ovarian cancer cells. Low expression of OLFM4 was also associated with high tumor FIGO stage and poor tumor differentiation. These results suggest *OLFM4* is downregulated by miR-486-5p, which contributes to ovarian cancer tumorigenesis. Conversely, estrogen receptor signaling downregulates miR-486-5p and upregulates OLFM4 expression, slowing the development and progression of ovarian cancer.

## INTRODUCTION

Ovarian cancer, together with endometrial cancer and cervical carcinoma, are the three most common gynecological malignant tumors [1]. Epithelium-derived ovarian serous tumors include benign serous cystadenoma, serous borderline tumors, and malignant serous adenocarcinoma. Ovarian serous cystadenomas are common ovarian lesions that may be precursors of serous borderline tumors, which can in turn progress to ovarian serous adenocarcinomas [2]. Ovarian serous adenocarcinoma accounts for about 75% of ovarian epithelial tumors and can be highly invasive. The ovaries secrete both estrogen and progesterone, and estrogens play a role in the development, growth, invasion and metastasis of ovarian tumors. Estrogen receptor alpha (ERα) induces gene expression changes in ovarian cancer cells [3]. High ERα expression is associated with reduced apoptosis in poorly-differentiated ovarian cancer [4]. However, patients with ERα*-*expression have a better prognosis [5]. The mechanisms of estrogens actions on the development and progression of gynecological tumors is not well understood.

Olfactomedin 4 (OLFM4), also known as hGC-1, GW112, hOlf D and pDP4 [6-9], was first cloned from myeloid precursor cells treated with granulocyte colony-stimulating factor [7]. OLFM4 is expressed mainly in the gastrointestinal tract, prostate, breast, bone marrow, and pancreas [7, 10]. OLFM4 expression is increased in cancers of the stomach, colon, pancreas, lung, and breast [11-13], and OLFM4 expression is correlated with tumor differentiation and prognosis. Patients with well-differentiated gastric cancer and higher OLFM4 expression have a five-year survival rate higher than patients with poorly differentiated cancer [14]. Induction of OLFM4 regulates adhesion and migration in colon cancer cells [15], promotes S phase transition and proliferation of pancreatic cancer cells [12], and inhibits migration and invasion of prostate cancer cells [16]. These data suggest that OLFM4 is involved in the pathogenesis and development of various tumors.

We previously demonstrated that aberrant OLFM4 expression also occurs in gynecological tumors. OLFM4 expression has been associated with progression of cervical intraepithelial neoplasia (CIN) and differentiation of cervical cancer [17]. Impairment of ERα-mediated OLFM4 expression promotes the malignant progression of endometrioid adenocarcinoma [18]. In a bioinformatics screen for molecules upstream of OLFM4, we discovered one potential regulator, miR-486-5p. There is a conserved miR-486-5p-binding site on the 3′ UTR of human *OLFM4* mRNA. miR-486-5p is enriched in muscle tissue [19], and miR-486-5p targets OLFM4 thereby playing a tumor-suppressive role in gastric cancer [20]. Up-regulation of miR-486-5p also contributes to the progression of pancreatic ductal adenocarcinoma [21]. In the current study, we investigated the expression of OLFM4, ERα, and PR in formalin-fixed, paraffin-embedded (FFPE) ovarian tissues, analyzed the expression of miR-486-5p in ovarian tissues and its association with OLFM4 levels and ERα signaling, examined the effects of OLFM4 on ovarian carcinoma cells, and evaluated the role of OLFM4 in the development and progression of ovarian cancer.

## RESULTS

### Ovarian serous adenocarcinoma tissues have aberrant olfactomedin 4 expression

**Figure 1:**
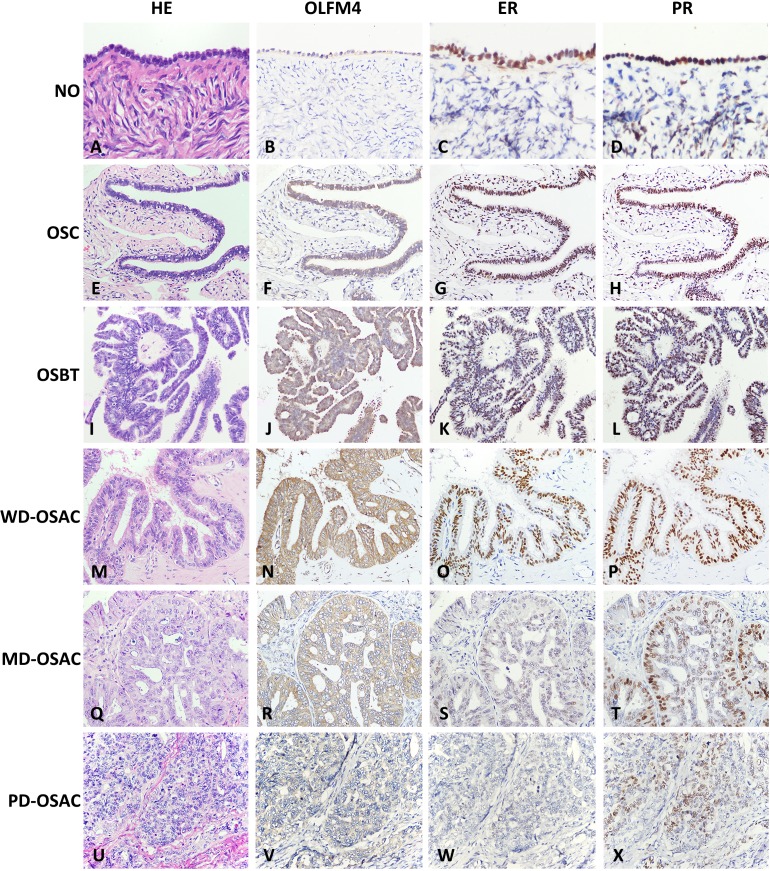
Expression of Olfactomedin 4 (OLFM4), estrogen receptor-a (ERα) and progesterone receptor (PR) in normal ovary (NO); ovarian serous cystadenoma (OSC); ovarian serous borderline tumor (OSBT); well-differentiated ovarian serous adenocarcinoma (WD-OSAC), moderately-differentiated ovarian serous adenocarcinoma (MD-OSAC) and poorly-differentiated ovarian serous adenocarcinoma (PD-OSAC) detected by immunohistochemistry Haematoxylin-eosin (HE), Morphology of NO **A.** OSC **E.** OSBT **I.**, and WD-OSAC **M.**, MD-OSAC **Q.** and PD-OSAC (U) stained by haematoxylin-eosin. OLFM4, OLFM4 staining was undetectable in NO **B.**. Immunoreactivity of OLFM4 gradually increased from OSC **F.** OSBT **J.** to WD-OSAC **N.**, and gradually decreased with lower degrees of differentiation in serous adenocarcinoma (N, R, V). ER, Immunoreactivity of ER gradually decreased from NO **C.** OSC **G.** OSBT **K.** to WD-OSAC **O.** ER staining was hardly detectable in MD-OSAC **S.** and PD-OSAC **W.** PR, Immunoreactivity of PR gradually decreased from NO **D.** OSC **H.** OSBT **L.**, WD-OSAC **P.**, MD-OSAC **T.** to PD-OSAC **X.**

To investigate whether olfactomedin 4 (OLFM4) is associated with ovary tumorigenesis, we examined its expression in normal ovary; serous cystadenoma; serous borderline tumor; and well-, moderately- and poorly-differentiated serous adenocarcinoma using immunohistochemistry (IHC). OLFM4 was stained in the cytoplasm of epithelial cells (Figure 1). The rates of OLFM4 high-expression in these tissues were 16.7, 34.3, 65.1, 72.2, 50.0, and 11.9%, respectively (Table S1 and Table S2). OLFM4 expression in well-differentiated serous adenocarcinoma was higher than normal ovarian epithelium (*P* < 0.001, Table S1), indicating an association of OLFM4 expression with ovarian tumorigenesis. Staining intensity of OLFM4 decreased along with the degree of differentiation of serous adenocarcinoma (Figure 1N, 1R, 1V). There was also a difference in OLFM4 expression between well- and poorly-differentiated serous adenocarcinomas (*P* < 0.001, Table S2).

### Olfactomedin 4 inhibits proliferation, metastasis, and invasion of ovarian serous adenocarcinoma cells

**Figure 2:**
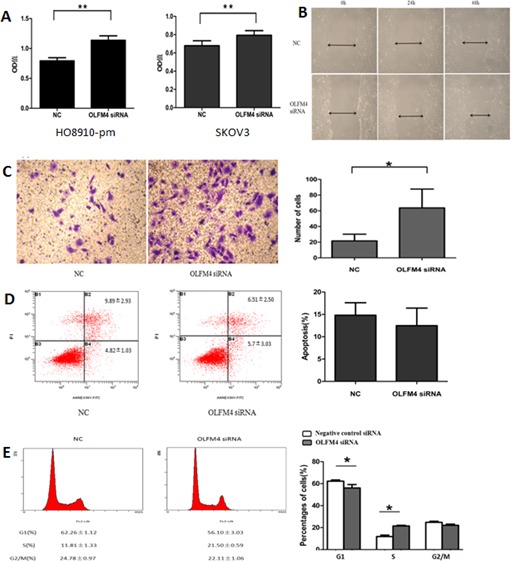
Effects of OLFM4 on ovarian serous adenocarcinoma cells **A.** Effects of OLFM4 on proliferation of HO8910-pm and SKOV3 measured by MTT (**P < 0.01). **B.** OLFM4 knockdown promoted migration of HO8910-pm cells by scratch wound assay. **C.** OLFM4 knockdown promoted invasion of HO8910-pm cells (*P < 0.05) by transwell invasion assay. **D.** Treatment of HO8910-pm cells with OLFM4 siRNA had no effect on cell apoptosis. **E.** Knockdown of OLFM4 in HO8910-pm cells transfected with specific siRNA resulted in decrease in G1 phase cells and increase in S phase cells (*P < 0.05).

In a previous study we demonstrated that knockdown of OLFM4 enhances the proliferation, migration, and invasion of endometrial carcinoma cells [18]. We therefore sought to investigate the effects of OLFM4 on ovarian serous adenocarcinoma cells. siRNA knockdown of *OLFM4* promoted cell proliferation in both HO8910-pm and SKOV3 cells (Figure 2A), but had no effect on cell apoptosis (Figure 2D). Knockdown of *OLFM4* in HO8910-pm cells increased cell migration (Figure 2B) in a scratch wound assay. In a transwell migration assay, the mean number of invaded cells per one visual field (at 200X magnification) was greater with *OLFM4* knockdown (mean number = 68) compared with the control group (mean number = 24) (Figure 2C). These results suggest that OLFM4 regulates ovarian serous adenocarcinoma cell proliferation and migration. Next, we examined the effects of OLFM4 on cell cycle progression using flow cytometry. HO8910-pm cells with *OLFM4* knockdown had decreased numbers of cells in G1 phase and increased numbers in S phase compared with control cells, demonstrating that OLFM4 facilitates cell cycle arrest at G1 (Figure 2E).

### OLFM4 expression is associated with the progression and differentiation of ovarian serous adenocarcinoma

**Figure 3:**
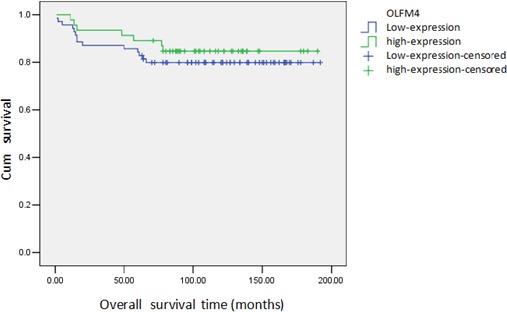
Cumulative survival curve of patients with ovarian serous adenocarcinoma and expression of OLFM4

Based on the expression of OLFM4 measured in paraffin-embedded tissues, we analyzed the association of OLFM4 expression with clinicopathological features and prognosis of ovarian serous adenocarcinoma. Low expression of OLFM4 was associated with high tumor FIGO stage and poor tumor differentiation, and not associated with patients' age or metastasis (Table 1). Of the 116 ovarian serous adenocarcinoma tissues, 21 patients died and the cumulative survival rate was 81.9%. The cumulative survival rate was 84.8% in 46 cases with high expression of OLFM4, and 80.0% in patients with low-expression of OLFM4. Though this difference is not statistically significant, the survival curve is straighter for patients with high OLFM4 expression (Figure 3).

**Table 1:** Association of OLFM4 expression detected by IHC with clinicopathological features of ovarian serous adenocarcinoma

Parameters	OLFM4 expression		*P*
	High	Low	
Age			
>50 (n=54)	21	33	0.875
≦50 (n=62)	25	37	
FIGO stages			
I-II (n=44)	23	21	0.030
III-IV (n=72)	23	49	
Histological grade			
Well (n=18)	13	5	<0.371
Moderate (n=56)	28	28	
Poor (n=42)	5	37	
Metastasis			
No (n=23)	11	12	0.371
Yes (n=93)	35	58	

High: High-expression; Low: Low-expression.

### OLFM4 expression is regulated by ERα signaling

Estrogen receptor signaling regulates the expression of OLFM4 in endometrioid adenocarcinoma [18]. To investigate whether this regulation also occurs in ovarian serous adenocarcinoma, we examined the expression of ERα and PR in ovarian tissues using IHC (Figure 1). The rates of ERα high-expression in normal ovary, serous cystadenoma, serous borderline tumor, and ovarian serous adenocarcinoma were 61.1, 45.7, 34.9 and 16.4%, respectively (Table S3). High-expression rates of PR in these tissues were 72.2, 60.0, 65.1 and 12.1%, respectively (Table S3). Expression of OLFM4 in ovarian serous adenocarcinoma was positively correlated with the expression of ERα but not PR (Table 2).

**Table 2:** Co-relationship of OLFM4 level with ERα and PR expression in ovarian tissues based on IHC data

OLFM4	NO (n=18)		OSC (n=35)		OSBT (n=43)		OSAC (n=116)	
4	High	Low	High	Low	High	Low	High	Low
ERα								
High	3	0	6	6	9	19	13	33
Low	8	7	10	13	6	9	6	64
*P*	0.130		0.713		0.606		0.005	
r	-		-		-		0.260	
PR								
High	2	1	8	4	17	11	8	38
Low	11	4	13	10	11	4	6	64
*P*	0.814		0.561		0.408		0.154	
r	-		-		-		-	

NO: normal ovary; OSC: ovarian serous cystadenoma; OSBT: ovarian serous borderline tumor; OSAC: ovarian serous adenocarcinoma. High: high-expression; Low: low-expression.

**Figure 4:**
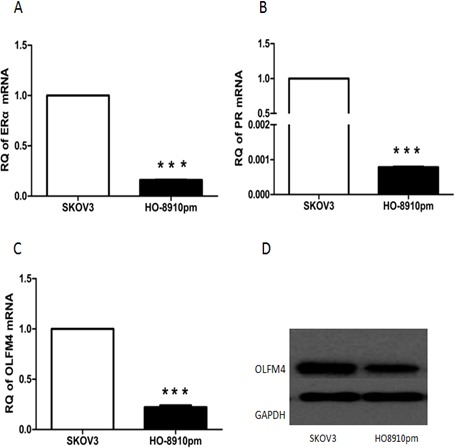
Expression of ERα **A.** PR **B.** OLFM4 **C.** mRNAs, and OLFM4 protein **D.** in ovarian serous carcinoma cells, ***P < 0.001.

**Figure 5:**
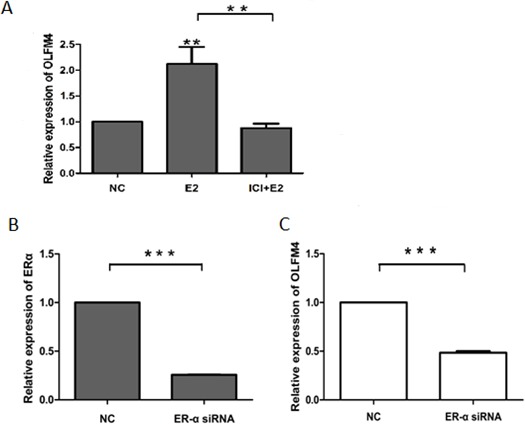
ERα-mediated regulation of OLFM4 expression in HO8910-pm cells **A.** E2 induced the expression of OLFM4 and estrogen receptor antagonist ICI 182 780 attenuated the OLFM4 mRNA increase induced by E2. **B.** Knockdown of ERα expression with siRNA. **C.** Down-regulation of ERα with siRNA reduced the E2-induced expression of *OLFM4*. ***P < 0.001, **P < 0.01. NC: negative control.

Next, we used ovarian cancer cells, SKOV3 and HO8910-pm, to investigate the association of OLFM4 expression with estrogen receptor signaling. Both SKOV3 and HO8910-pm cells expressed OLFM4, ERα, and PR. OLFM4 and ERα expression in SKOV3 cells, a moderately-differentiated ovarian serous adenocarcinoma cell line, was higher than the expression in HO8910-pm, a poorly-differentiated ovarian serous adenocarcinoma cell line (Figure 4), further suggesting the correlation of OLFM4 expression with ERα expression and degree of tumor differentiation. Stimulation of cells with 17β-estradiol (E2) increased the production of OLFM4 mRNA and protein in HO8910-pm cells, while an estrogen receptor antagonist, ICI 182 780, attenuated the *OLFM4* mRNA increase induced by E2 (Figure 5A). Knockdown of ERα reduced the E2-induced expression of *OLFM4* mRNA in HO8910-pm cells (Figure 5B, 5C). These results suggest that ERα-mediated signaling enhances expression of OLFM4. Thus, estrogen receptor signaling in the development of ovary serous adenocarcinoma may be partially due to the regulation of *OLFM4*.

### ERα-mediated miR-486-5p regulates expression of OLFM4

**Figure 6:**
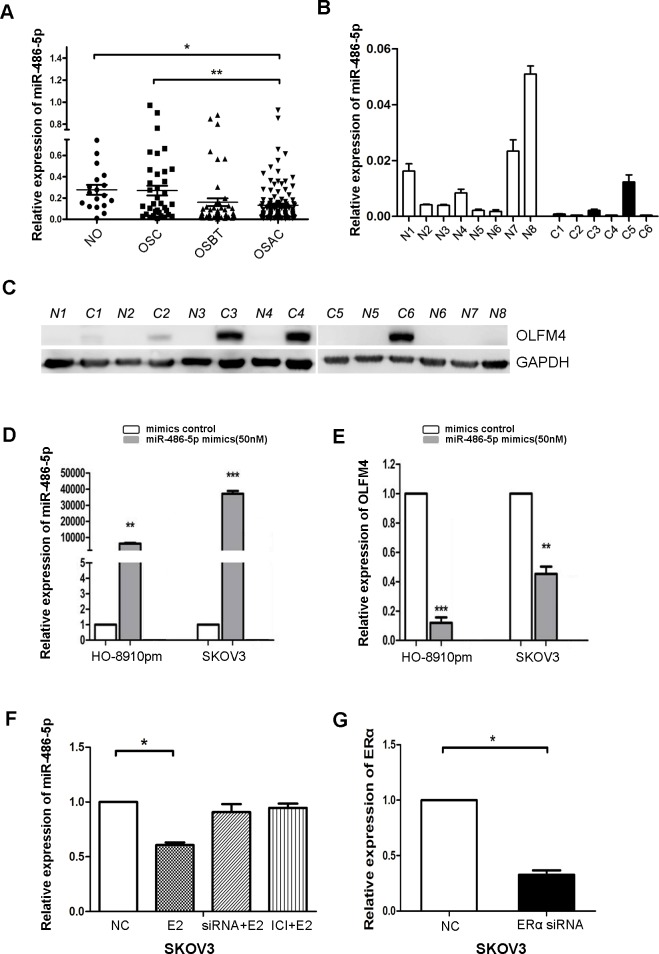
Estrogen regulates the expression of miR-486-5p, which targets OLFM4 **A.** Comparison of miR-486-5p levels among formalin-fixed, paraffin-embedded (FFPE) tissues of normal ovary (NO), ovarian serous cystadenoma (OSC), ovarian serous borderline tumor (OSBT), and ovarian serous adenocarcinoma (OSAC) detected by IHC. **B.** miR-486-5p expression in frozen fresh ovarian serous adenocarcinoma and normal tissues detected by real-time PCR. C and N represent individual tissues of serous adenocarcinoma and normal ovary, respectively. **C.** OLFM4 expression in frozen fresh ovarian serous adenocarcinoma and normal tissues was detected by Western blot. **D.** Transfection of miR-486-5p mimics increased miR-486-5p levels in ovarian cancer cells. **E.** Increased miR-486-5p led to reduced *OLFM4* mRNA levels. **F.** Treatment with estrogen (E2) resulted in decreased miR-486-5p levels. Treatment with estrogen receptor antagonist ICI 182 780 (ICI) or knockdown of ERα with ERα-specific siRNA attenuated E2-induced decrease of miR-486-5p levels in SKOV3 cells. **G.** Knockdown of ERα expression with ERα-specific siRNA in SKOV3 cells. NC: negative control, ***P < 0.001, **P < 0.01, and *P < 0.05.

Bioinformatics analysis identified a putative miR-486-5p target site on the 3′ UTR sequence of human *OLFM4* [20]. miR-486-5p levels were measured in FFPE tissues of normal ovary, ovarian serous cystadenoma, ovarian serous borderline tumor, and ovarian serous adenocarcinoma by real-time PCR. There was a difference in miR-486-5p levels between normal ovary and ovarian serous adenocarcinoma (Figure 6A). To compare with the OLFM4 expression data measured by IHC, the average value of miR-486-5p in normal tissues was calculated and all other samples were then calibrated to normal tissues. A relative value above the average level in normal tissues was assigned as miR-486-5p high expression, and vice-versa. Rates of miR-486-5p high expression in ovarian serous cystadenoma, ovarian serous borderline tumor, and ovarian serous adenocarcinoma tissues were 42.9, 32.6 and 25.9%, respectively (Table S4). miR-486-5p expression was decreased in ovarian serous adenocarcinoma as compared to normal ovary, and inversely correlated with OLFM4 expression in ovarian serous adenocarcinoma (Table 3).

**Table 3:** Co-relationship of OLFM4 level with miR-486-5p expression

OLFM4	OSC (n=35)		OSBT (n=43)		OSAC (n=116)	
	High	Low	High	Low	High	Low
miR-486-5p						
High	5	7	10	18	7	39
Low	10	13	4	11	23	47
*P*	0.713		0.606		0.034	
r	-		-		−0.197	

OSC: ovarian serous cystadenoma; OSBT: ovarian serous borderline tumor;

OSAC: ovarian serous adenocarcinoma. High: high-expression; Low: low-expression.

When OLFM4 expression was analyzed by Western blot and miR-486-5p expression was measured by real-time PCR in frozen fresh human ovarian cancer and normal tissues, we found that miR-486-5p levels in ovarian cancer tissues were lower than in normal tissues (Figure 6B) and that OLFM4 was more frequently detectable in ovarian cancer tissues as compared with normal tissues (Figure 6C).

To investigate whether OLFM4 expression is regulated by miR-486-5p, miR-486-5p was overexpressed in adenocarcinoma cells. HO8910-pm and SKOV3 cells transfected with miR-486-5p mimics, had decreased OLFM4 mRNA levels, indicating that OLFM4 is a potential target of miR-486-5p in ovarian serous adenocarcinoma (Figure 6D, 6E). Interestingly, when ovarian cancer cells were treated with E2, miR-486-5p expression was reduced (Figure 6F). The estrogen receptor antagonist ICI 182 780 or knockdown of ERα attenuated E2-induced changes in miR-486-5p expression in SKOV3 cells (Figure 6F), supporting a potential inhibitory effect of ERα signaling on miR-486-5p expression.

## DISCUSSION

Aberrant expression of OLFM4 has been observed in some cancerous tissues, especially in those of the digestive system. In our previous studies we demonstrated that OLFM4 is also functionally associated with gynecological tumors such as cervical cancer and endometrial adenocarcinoma [17, 18]. In the current study, aberrant OLFM4 expression was observed in ovarian serous adenocarcinoma tissues. Mechanistic studies demonstrated estrogen receptor-mediated miR-486-5p targeting of OLFM4 in ovarian cancer

Scratch wound and transwell migration assays indicated that OLFM4 inhibits cell migration and invasion of ovarian cancer cells (Figure 2B, 2C). However, expression of OLFM4 was not associated with metastasis in our clinicopathological analysis (Table 1). This discrepancy is probably due to small sample sizes. There were 58 patients with low OLFM4 expression in a total 93 metastasis patients, but only 35 patients with high OLFM4 expression. Although an association was not observed, there was a tendency for patients with low OLFM4 expression to be prone to metastasis.

Both up- and down-regulation of miR-486 have been associated with the development and progression of tumors. Down-regulation of miR-486 contributes to the progression and metastasis of breast, liver and lung cancers [22-24] and up-regulation of miR-486 promotes the progression of pancreatic ductal adenocarcinoma [21] and gliomas [25]. miR-486 targets Pim-1 kinase in lung cancer [26], OLFM4 in gastric cancer [20], CLDN10 and CITRON in hepatocellular carcinoma [24], and plasminogen activator inhibitor-1 in human myxoid liposarcoma [27]. Although miR-486 is involved in many tumors and targets different effecter molecules, our results support the idea that OLFM4 is one of potential miR-486 targets.

Expression of ERα is associated with estrogen-dependent growth, invasion, and response to endocrine therapy in ERα-positive ovarian cancer, but the exact mechanism remains less clear. Low concentrations of estrogen do not affect the proliferation of ovarian cancer cells. In contrast, high concentrations of estrogen inhibits survival rate of ovarian cancer cells [28]. A combination of estrogen and progesterone decreases cell proliferation and inhibits the expression of Bcl-2 *via* let-7a and miR-34b in ovarian cancer cells [28]. miR-206 inhibits cellular proliferation and invasion of ERα-positive ovarian cancer cells [29]. The phosphoinositide 3-kinase (PI3K)/AKT pathway is involved in estrogen-induced metastasis in ovarian cancer cells [30]. *OLFM4* is regulated by estrogen in the human endometrium [31]. Here we demonstrated that ERα-mediated signaling regulates the expression of miR-486-5p, which targets *OLFM4* in ovarian cancer. Our study suggests that the role of ER signaling in ovarian cancer may be partially due to the regulation of mi-486-5p and *OLFM4*.

## MATERIALS AND METHODS

### Subjects and tissue samples

Formalin-fixed, paraffin-embedded (FFPE) tissues of ovarian serous adenocarcinoma (*n* = 116; mean patient age = 50) and corresponding patient clinicopathological data were collected from December 1998 to December 2008 at the Department of Pathology, First Affiliated Hospital of Sun Yet-sen University. Patients were followed up until 1 June 2014 or until death. All specimens were obtained from surgery. None had received pre-operative radiotherapy, chemotherapy or hormone drug therapy. Patients were evaluated in accordance with the International Federation of Gynecology and Obstetrics (FIGO) criteria for staging ovarian cancers. The number of cases classified as FIGO stage I, II, III and IV were 22, 22, 59 and 13, respectively. Histological grade was determined in accordance with the World Health Organization (WHO) grading system. The number of cases classified as WHO grade 1, 2 and 3 were 18, 56 and 42, respectively. FFPE specimens of normal ovary (*n* = 18), ovarian serous cystadenoma (*n* = 35), and ovarian serous borderline tumor (*n* = 43) were also collected. Frozen fresh tissues of well-differentiated ovarian serous adenocarcinoma (*n* = 6) and normal ovary (*n* = 8) were collected.

### Steroid hormones and chemicals

17β-estradiol (E2) was purchased from Sigma-Aldrich (St. Louis, MO, USA). ICI 182 780 was purchased from Tocris Cookson Ltd (Bristol, UK).

### Immunohistochemistry

Immunohistochemistry (IHC) was conducted as described previously with antibodies to OLFM4 (Lifespan, rabbit anti-human polyclonal, diluted at 1/100; Seattle, USA), ERα (Kit-0012-2, rabbit anti-human monoclonal, ready to use; Maixin Biotechnology, Fuzhou, Fujian, China), PR (Kit-0013-2, rabbit anti-human monoclonal, ready to use; Maixin Biotechnology), and horseradish peroxidase-labeled secondary antibody (Maixin Biotechnology) in accordance with manufacturer's instructions. Color was developed with diaminobenzidine (Dako Corp, Carpinteria, CA, USA) incubated for 5-10 min at room temperature. Slides were counterstained with haematoxylin and examined by light microscopy.

Staining intensity was graded according to the following criteria described in our previous study [18]: 0 (no staining), 1 (weak staining, light yellow), 2 (moderate staining, yellow with brown), and 3 (strong staining, brown). The percent staining was graded according to the proportion of positive stained cells as follows: 0 for ≤5% positive cells; 1 for 6-25% positive cells; 2 for 26-50% positive cells and 3 for ≥51% positive cells. The immunoreactive score (IRS) was used to evaluate results. IRS = staining intensity × percent of positive cells. An IRS score of 4 and higher was regarded as high expression.

### Cell culture

SKOV3, a moderately-differentiated ovarian serous adenocarcinoma cell line, was purchased from Shanghai Institute for Biological Sciences (Shanghai, China). HO8910-pm, a poorly-differentiated ovarian serous adenocarcinoma cell line, was reserved in Department of Gynecology, the First Affiliated Hospital, Sun Yat-sen University. The cells were cultured in DMEM (Hyclone Laboratories) supplemented with 10% fetal bovine serum (GIBCO, Australia), 100 U/ml penicillin G and 100 μg/ml streptomycin in a humidified atmosphere of 5% CO_2_, at 37°C.

### Cell transfection

SKOV3 and HO8910-pm cells were seeded in 6-well plates for 24 hrs before transfection. Cells grown to 30-50% confluence were transfected using lipofectamine RNAiMAX (Invitrogen) with one of small interfering RNA (siRNA) duplexes specific for human ERα and OLFM4, and miR-486-5p mimics or corresponding negative control siRNA or negative control mimics according to the manufacturer's protocol. The specificity and efficacy of siRNA or miRNA were initially evaluated.

### Cell proliferation assay

Cell proliferation was estimated using 3-(4,5-dimethylthiazol-2-yl)-2,5-diphenyltetrazolium bromide (MTT; Sigma-Aldrich). 9×10^3^ cells per well were seeded in 96-well plates. Cells grown to 30-50% confluence were transfected with siRNA specific for OLFM4 or negative control siRNA for 72 hrs. At the end of the treatment, 10ul of MTT solution (5mg/ml in phosphate-buffered saline) was added to the medium and the cells were incubated at 37°C for 4 hrs. The MTT-containing medium was removed, and the cells were lysed with 200ul dimethyl sulfoxide for 20 min. Absorbance values were measured using a microplate reader at 570 nm, and the results were plotted as mean ± SD.

### Cell apoptosis assay

3×10^5^ cells were pelleted by centrifugation, washed twice with cold PBS, and resuspended in 500 μl cold Annexin binding buffer containing 5 μl Annexin V-FITC and 5 μl propidium iodide. The cells were incubated for 15 minutes in the dark at room temperature and analyzed using a Becton Dickinson FACScan (Becton Dickinson Immunocytometry Systems, San Jose, CA). The experiment was performed in triplicate, independently.

### Cell cycle analysis

3×10^5^ cells were washed twice with cold PBS and fixed with 70% ethanol overnight. Cells were washed with PBS, pelleted by centrifugation, and resuspended in 100 ul RNase A (100ug/ml) and incubated at 37°C for 30 min. With an addition of 400ul propidium iodide, cells were then incubated for 15 minutes in the dark at room temperature and analyzed with flow cytometry.

### Scratch wound assay

Cells were plated in 6-well plates and incubated overnight until 30%-50% confluent, then transfected with 100 nM OLFM4 siRNA or negative control siRNA. Confluent cells wounded by scratching with a 20 ul micropipette tip. Wells were washed three times with PBS to remove dislodged cells. ‘Wound closure’ was monitored at 0, 24, 48 hrs and photographed through an inverted microscope.

### Transwell migration and invasion assays

4×10^4^ cells were plated in serum-free media in the upper chamber of 24-well Transwell Chambers (Corning Incorporated, Life Sciences), while media containing 10% FBS were added to the lower chamber as chemoattractant. The cells were incubated at 37°C in a 24-well plate and allowed to invade through the matrigel (BD Biosciences) for 36 hrs. Cells on the upper surface of the filters were gently removed by wiping with a cotton swab. The cells under the surface of the lower chamber were fixed in 4% paraformaldehyde, stained with 1% crystal violet and counted (Ten random 200× fields per well). The mean number of cells per field of view was calculated.

### Western blot analysis

Total cell proteins were isolated with a total protein extraction kit (Keygen). Concentration of proteins was determined with the BCA protein assay kit (Cowin BioTech, Beijing, China). 30μg of protein was loaded and separated in 10% sodium dodecyl sulfate polyacrylamide gel electrophoresis (SDS-PAGE) gel and transferred to polyvinylidine difluoride membranes (Millipore, Bedford, MA). The antibody against OLFM4 (Lifespan, rabbit anti-human polyclonal, Seattle, USA) was used to analyze protein expression. Proteins were visualized using horseradish peroxidase-conjugated secondary antibody. Signal was detected by enhanced chemoluminescence techniques (Millipore). Detection of GAPDH with specific antibody (Cell Signaling Technology) was used as the loading control.

### Quantitative real-time reverse transcription PCR

Total RNA and miRNA were prepared from cultured cells or paraffin-embedded tissues, respectively, using Trizol reagent (Invitrogen Life Technology) according to the manufacturer's protocol. 1000 ng of total RNA was converted to cDNA with a First Strand cDNA Synthesis Kit (Toyobo, Osaka, Japan). Reactions were conducted in a 20 ul reaction volume in triplicate using FastStart Universal SYBR Master (Rox; Roche, Mannheim, Germany). SYBR PrimeScript miRNA RT-PCR Kit (Takara, Japan) was used for real-time PCR reaction of miRNA. Expression fold-change of genes was evaluated using 2^−^ΔΔ^Ct^. PCR primer sequences included *OLFM4* sense ACTGTCCGAATTGACATCATGG, antisense TTCTGAGCTTCCACCAAAACTC; *ESR1* sense GGTGCCCTACTACCTGGAGAA, antisense GCCATACTTCCCTTGTCATTG. *PGR* sense ACACCTCCAGTTCTTTGCTGAC, antisense ATTCTTTCATCCGCTGTTCATT; *GAPDH* sense AGAAGGCTGGGGCTCATTTG, antisense AGGGGCCATCCACAGTCTTC; miR-486-5p sense 5′-GTACTGAGCTGCCCCGAGAAA-3′, antisense Uni-miR qPCR Primer; U6 sense Universal_ RNU6B _ Primer, and antisense Uni-miR qPCR Primer.

### Statistical analyses

Statistical analyses were performed using GraphPad Prism 5.0 software. Student's t-test or ANOVA was used for statistical comparisons of quantitative data. The differences of OLFM4, ERα and PR expression among multiple groups were analyzed by a chi-square test, Fisher's exact test, or the Wilcoxon rank sum test. Associations between OLFM4 expression and ERα or PR and between OLFM4 and miR-486-5p were analyzed by Pearson chi-squared test or Spearman rank correlation test. The relationships between OLFM4 expression and clinico-pathological characteristics were analyzed by the chi-squared test. The Kaplan-Meier method was used to calculate the survival curve. A log-rank test was used for univariate survival analysis. A *P* < 0.05 was considered statistically significant. When comparing two groups, the Bonferroni method was used to adjust the inspection standard.

## SUPPLEMENTARY MATERIAL TABLES


