# Supplementary material for: Estrogen receptor-mediated miR-486-5p regulation of OLFM4 expression in ovarian cancer
Source: Oncotarget. 2016 Feb 7;7(9):10594–605. doi: 10.18632/oncotarget.7236 (PMC4891143; doi:10.18632/oncotarget.7236)
Supplement: Supplementary file 1 [file oncotarget-07-10594-s001.pdf]

Estrogen receptor-mediated miR-486-5p regulation of OLFM4 expression in ovarian cancer

Supplementary Material

Table S1 OLFM4 expression in ovarian tissues detected by IHC

Tissues	OLFM4	
	High (%)	Low (%)
NO	3/18 (16.7)	15/18 (83.3)
OSC	12/35 (34.3)	23/35 (65.7)
OSBT	28/43 (65.1)	15/43 (34.9)
WD- OSAC	13/18 (72.2)	5/18 (27.8)
P	<0.001	

NO: normal ovary; OSC: ovarian serous cystadenoma; OSBT: ovarian serous borderline tumor; WD-OSAC: well-differentiated ovarian serous adenocarcinoma. High: high-expression; Low: low-expression.

Table S2 OLFM4 expression in ovarian serous adenocarcinomas with different levels of differentiation

Serous adenocarcinomas	OLFM4	
	High (%)	Low (%)
Well-differentiated[①]	13/18 (72.2)	5/18 (27.8)
Moderately-differentiated[②]	28/56 (50.0)	28/56 (50.0)
Poorly-differentiated[③]	5/42 (11.9)	37/42 (88.1)
P	<0.001	

[①-②]P =0.034； [①-③]P=0.000； [②-③]P=0.000. Bonferroni test was used for multiple comparisons between each two groups and the significant level was adjusted to 0.05/3=0.017. High: high-expression; Low: low-expression.

Table S3 ERα and PR expression in ovarian tissues detected by IHC

Tissues	ERα		PR	
	High (%)	Low (%)	High (%)	Low (%)
NO	11/18 (61.1)	7/18 (38.9)	13/18 (72.2)	5/18 (27.8)
OSC	16/35 (45.7)	19/35 (54.3)	21/35 (60.0)	14/35 (40.0)
OSBT	15/43 (34.9)	28/43 (65.1)	28/43 (65.1)	15/43 (34.9)
OSAC	19/116 (16.4)	97/116 (83.6)	14/116 (12.1)	102/116 (87.9)
P	<0.001		<0.001	

NO: normal ovary; OSC: ovarian serous cystadenoma; OSBT: ovarian serous borderline tumor; OSAC: ovarian serous adenocarcinoma. High: high-expression; Low: low-expression.

Table S4 miR-486-5p expression in ovarian tissues detected by real-time PCR

Tissues	miR-486-5p	
	High (%)	Low (%)
NO	-	-
OSC	15/35 (42.9)	20/35 (57.1)
OSBT	14/43 (32.6)	29/43 (67.4)
OSAC	30/116 (25.9)	86/116 (74.1)
P	<0.001	

NO: normal ovary; OSC: ovarian serous cystadenoma; OSBT: ovarian serous borderline tumor; OSAC: ovarian serous adenocarcinoma. High: high-expression; Low: low-expression.
